# Lymphoepithelioma-like hepatocellular carcinoma: a case report and a review of the literature

**DOI:** 10.1186/1477-7819-11-97

**Published:** 2013-05-04

**Authors:** Masahiro Shinoda, Yoshie Kadota, Hanako Tsujikawa, Yohei Masugi, Osamu Itano, Akihisa Ueno, Kisho Mihara, Taizo Hibi, Yuta Abe, Hiroshi Yagi, Minoru Kitago, Shigeyuki Kawachi, Akihiro Tanimoto, Michiie Sakamoto, Minoru Tanabe, Yuko Kitagawa

**Affiliations:** 1 Department of Surgery, School of Medicine, Keio University, 35 Shinanomachi, Shinjuku, Tokyo, 160-8582, Japan; 2 Department of Pathology, School of Medicine, Keio University, 35 Shinanomachi, Shinjuku, Tokyo, 160-8582, Japan; 3 Department of Diagnostic Radiology, School of Medicine, Keio University, 35 Shinanomachi, Shinjuku, Tokyo, 160-8582, Japan

**Keywords:** Lymphoepithelioma, Lymphoepithelioma-like carcinoma, Hepatocellular carcinoma

## Abstract

We report a rare case of lymphoepithelioma-like hepatocellular carcinoma. A 79-year-old Japanese man had undergone curative resection of extrahepatic bile ducts because of bile duct cancer 9 years prior. The bile duct cancer was diagnosed as mucosal adenocarcinoma, and the patient had been followed up every 6 months for the last 9 years. A recent computed tomography examination revealed a tumor, 4.2 cm in size, in the lateral segment of the liver. Based on the imaging findings, the tumor was diagnosed as hepatocellular carcinoma. Serology tests were negative for hepatitis B and C viruses. Chest and abdominal image analyses showed no evidence of metastasis, but a swollen lymph node was noted around the abdominal aorta. The patient subsequently underwent extended lateral segmentectomy and resection of the swollen lymph node. Microscopically, the tumor had the characteristic appearance of poorly differentiated hepatocellular carcinoma. Moreover, an abundant infiltration of inflammatory cells was observed in the tumor. Therefore, we diagnosed the tumor as lymphoepithelioma-like hepatocellular carcinoma. The resected para-aortic lymph node also had a carcinoma with features similar to those of the main tumor. The patient has been alive for 20 months since performance of the surgery. Since the first report of lymphoepithelioma-like hepatocellular carcinoma in 2000, only nine cases have been reported in the medical literature, and the clinicopathological features of the disease have not been well documented. Herein, we describe the clinicopathological features of this case for further understanding of the disease and review past cases in the literature.

## Background

Lymphoepithelioma is a form of undifferentiated carcinoma, characterized by prominent lymphoid stroma, and was originally described in the nasopharynx. Lymphoid stroma-rich carcinomas arising in other organs have been termed lymphoepithelioma-like carcinoma (LELC). Lymphoepithelioma and LELC arising in the salivary glands, stomach, lung, and thymus are often related to Epstein-Barr virus (EBV) infection. However, EBV is not usually found in LELC of the skin, breast, urinary bladder, and uterine cervix. In the hepatobiliary tract, primary LELCs are comparatively rare, and most have been identified as lymphoepithelioma-like cholangiocarcinomas (LEL-CCCs) [1-8]. According to the PubMed database of the National Library of Medicine, only nine cases of lymphoepithelioma-like hepatocellular carcinoma (LEL-HCC) in five reports have been described from 2000 to date [9-13]. Prior to 2000, there were some prior case reports in which the clinicopathological features of marked inflammatory cell infiltration in HCC were described, but the term LEL-HCC was not used [14,15]. It is not entirely clear whether the clinicopathological features of marked inflammatory cell infiltration in HCC cases reported before 2000 are distinguishable from those reported as LEL-HCC after 2000 [9-13]. The clinicopathological features were quite variable even among the nine cases reported as LEL-HCC. Herein, we report a unique case of HCC with marked inflammatory cell infiltration and, to further understand the disease’s characteristics, we review previous cases reported after the term LEL-HCC first appeared in 2000.

## Case presentation

**Figure 1:**
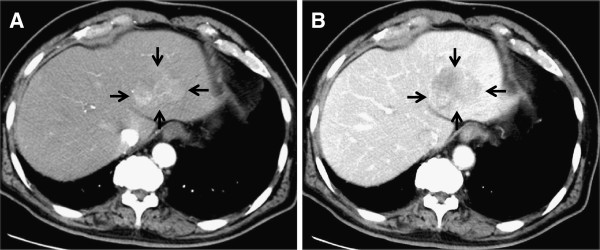
**Preoperative contrast-enhanced computed tomography findings.** Contrast-enhanced computed tomography revealed a relatively well-circumscribed mass, 42 mm in diameter, in the lateral segment of the liver. The tumor showed an enhancement in the arterial phase (**A**) and washout in the late phase (**B**).

**Figure 2:**
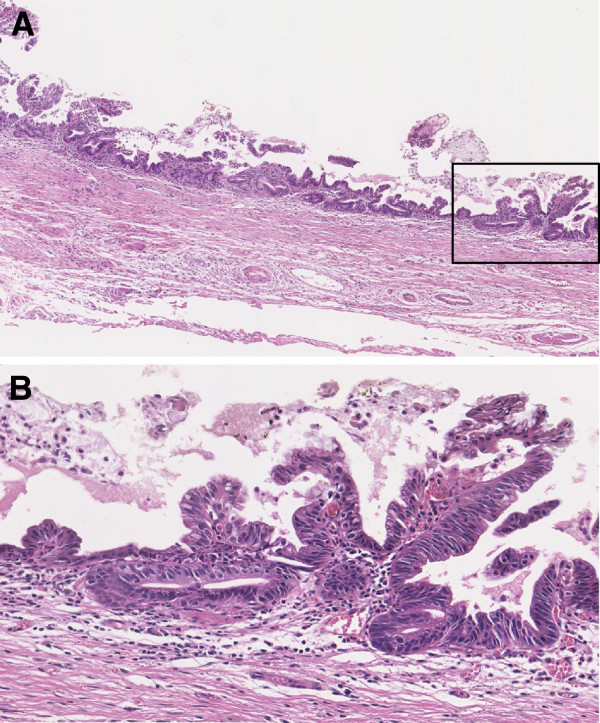
**Microscopic findings of the specimen of extrahepatic bile duct resected 9 years prior.** We examined the extrahepatic bile duct specimen resected 9 years prior and confirmed that it was well-differentiated adenocarcinoma limited to the mucosal layer of the extrahepatic bile duct throughout the specimen; hematoxylin-eosin, original magnification ×4 in (**A**) and ×20 in (**B**). A magnified observation of the rectangle in (**A**) is shown in (**B**).

A 79-year-old Japanese man had undergone complete extrahepatic bile duct excision and reconstruction of biliary-enteric continuity because of middle bile duct cancer 9 years prior. A pathological examination revealed that it was a well-differentiated adenocarcinoma limited to the mucosal layer, and the surgical margins were negative. The patient had been followed up every 6 months by blood analysis and imaging for the last 9 years. A recent contrast-enhanced computed tomography (CT) scan revealed a relatively well-circumscribed tumor, 42 mm in diameter, in the lateral segment of the liver. The tumor showed enhancement in the arterial phase and washout in the late phase (Figure 1A and 1B). Contrast-enhanced magnetic resonance imaging (MRI) with gadoxetic acid disodium (Gd-EOB-DTPA; Primovist®; Bayer Schering Pharma, Berlin, Germany) showed enhancement in the arterial phase and a defect in the hepatobiliary phase. The serum levels of alpha-fetoprotein (43 ng/ml; normal 0–20 ng/ml) and protein induced by vitamin K absence (PIVKA)-II (1957 mAU/ml; normal 0–39 mAU/ml) were elevated, although the levels of carcinoembryonic antigen and CA19-9 were within normal limits. We re-examined a specimen of extrahepatic bile duct that had been resected 9 years prior and confirmed that the well-differentiated adenocarcinoma was limited to the mucosal layer throughout the specimen (Figure 2A and 2B). The tumor was diagnosed as HCC because: (1) the imaging findings were compatible with HCC, (2) alpha-fetoprotein and PIVKA-II were elevated, (3) it was very unlikely that the past mucosal bile duct adenocarcinoma had metastasized to the liver, and (4) gastrointestinal endoscopy and colonoscopy did not find any primary diseases. Aspartate aminotransferase, alanine aminotransferase, alkaline phosphatase, gamma-glutamyl transferase, albumin, and total bilirubin levels were all within normal limits. Serology tests were negative for hepatitis B and C viruses. Abdominal CT and ultrasound scans revealed neither steatosis nor cirrhosis. A CT examination immediately prior to the operation revealed that the tumor had grown to 50 mm in diameter, but there was no evidence of metastasis except for a swollen para-aortic lymph node on the left side of the caudate lobe. Although the patient had taken two kinds of oral antiplatelet drugs because of a past coronary artery bypass graft, one of the drugs was temporarily discontinued as a precautionary measure for hemostatic safety. Extended lateral segmentectomy and resection of the swollen lymph node were subsequently performed 12 weeks after the tumor was discovered by CT. The postoperative course was uneventful, and the patient was discharged on postoperative day 19.

**Figure 3:**
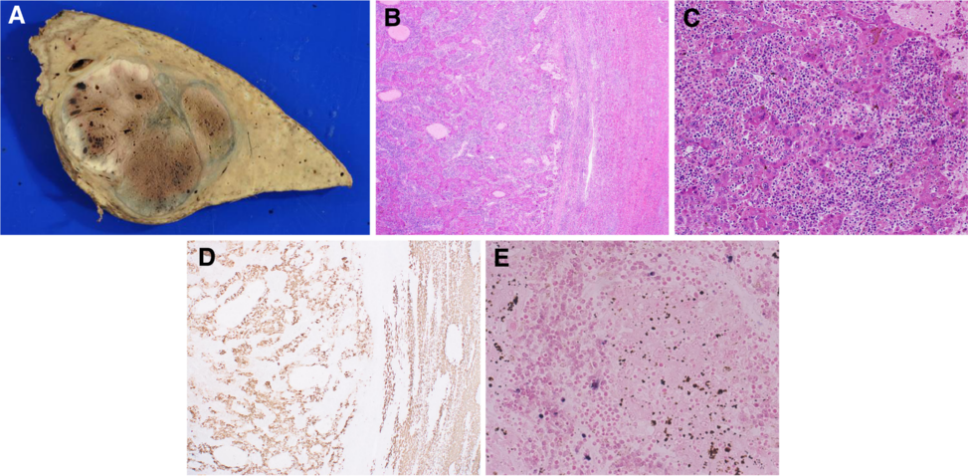
**Macroscopic and microscopic findings of the resected specimen.** (**A**) The gross appearance of the tumor (55 × 44 mm). The cut surface was yellow-grey-white in color admixed with brown focal spots. The tumor was surrounded by a thin fibrous capsule, and septum formation was also evident. The background liver was not cirrhotic. The brown focal spots recognized on this macroscopic cut surface proved to be periotic change on the microscopic examination. (**B**,**C**) The tumor was composed of solid syncytial nests of large malignant cells with pleomorphic and hyperchromatic nuclei, which were morphologically identical to poorly differentiated hepatocellular carcinoma. Abundant infiltration of small lymphocytes was observed in the background. The lymphocytes infiltrated even in the syncytial nests of hepatocellular carcinoma; hematoxylin-eosin, original magnification ×2 in **B** and ×20 in **C**. (**D**) Immunohistochemical staining for hepatocytes (×2). Both hepatocellular carcinoma cells and non-cancerous hepatocellular cells were positive for anti-hepatocyte. (**E**) The tumor cells were negative for Epstein-Barr virus as indicated by EBER *in situ* hybridization (original magnification ×40).

Macroscopically, the tumor, 55 × 44 mm in size, was a relatively well-circumscribed solid rubbery mass. The color of the cut surface was yellow-grey-white admixed with brown focal spots (Figure 3A). Microscopically, the tumor was identical to poorly differentiated HCC (Figure 3B and 3C). There were biliary and portal invasions. The areas with brown focal spots in the macroscopic examination corresponded periotic change microscopically. A dense infiltration of cells, most of which were lymphocytes, was observed all over the tumor. Therefore, we classified this HCC with heavy lymphoid infiltration as ‘LEL-HCC.’ Immunohistochemical analyses revealed that the HCC cells showed positivity for anti-hepatocyte (Figure 3D) but not for CK7 and CK19. The lymphocytes in the lymphoid stroma were composed of a mixture of CD3-positive T lymphocytes and L26-positive B lymphocytes. Lymphocyte infiltration was evident even in the cancer nests, and the cells were predominantly CD3-, CD5-, CD7-, CD8-, and TIA1-positive T lymphocytes. The carcinoma cells were negative for EBV as shown by EBER *in situ* hybridization (Figure 3E). A carcinoma was also present in the resected para-aortic lymph node, and its features were similar to those of the primary tumor. A few scattered atypical small bile ducts were observed in the resected liver, and they showed severe atypia, suggestive of well-differentiated adenocarcinoma *in situ*.

**Figure 4:**
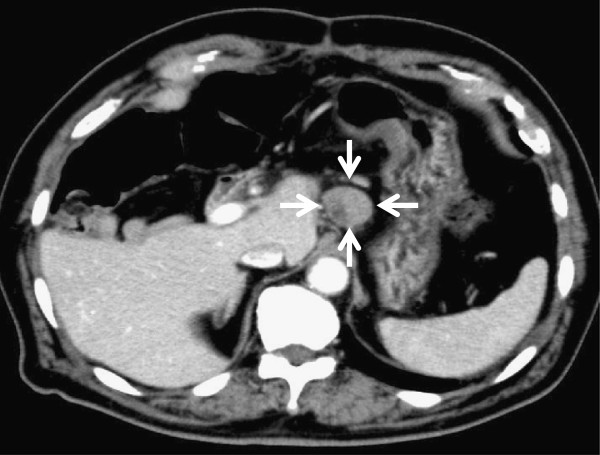
**Postoperative contrast-enhanced computed tomography findings.** Six months after surgery, a computed tomography examination revealed multiple swollen lymph nodes around the abdominal aorta. One of the para-aortic lymph nodes located at the left side of the caudate lobe is indicated by arrows.

Six months after surgery, a CT examination revealed multiple swollen lymph nodes around the abdominal aorta (Figure 4). Because it was very unlikely that the past mucosal bile duct adenocarcinoma metastasized to the lymph node and no primary malignant disease has been found, we initially suspected metastases from the resected HCC. We were also concerned about the potential existence of undiscovered intrahepatic bile duct cancer in the remaining liver because of the finding of severe atypia in the bile ducts on the resected side. We attached much more importance to the fact that the resected para-aortic lymph node had similar features to the primary tumor than to the finding of severe atypia in the resected side, and we concluded that the multiple swollen lymph nodes were metastases from the HCC. The patient started on oral sorafenib and is still alive 20 months postoperatively.

## Discussion

Lymphoepithelioma is an undifferentiated carcinoma with prominent lymphoid stroma, originally described in the head and neck region by Regaud and Schminke in 1921 [16]. Lymphoid stroma-rich carcinomas arising in other organs are called LELCs and have been reported in a variety of anatomic sites including the skin [17-20], lacrimal and salivary glands [21,22], thyroid gland [23], thymus [24], breast [25], lung [26], esophagus [27], stomach [28], colon [29], renal pelvis [30-33], ureter [32], kidney [31], bladder [34], prostate [31], uterine cervix [35], vulva [36,37], and vagina [38]. LELC in the liver has also been reported, but most of these cases have instead been referred to as LEL-CCCs [1-8]. Shirabe and colleagues reported a case of HCC with lymphoid stroma in 1995 and suggested that this disease could be a distinct category with a more favorable prognosis [14]. Wada and associates surveyed 163 patients with HCC and found that 11 patients had clinicopathological features of marked inflammatory cell infiltration [15]. Although both of these reports might have recognized clinicopathological features that are characteristic of LEL-HCC, they did not use this term. LEL-HCC appears to have been first described in 2000 by Emile and coworkers, who surveyed 162 HCCs in explanted livers and found 5 cases with abundant lymphoid stroma [9]. Subsequently, Szekely reported that the tumors were indistinguishable from lymphoepithelioma-like carcinoma [39]. Since these five cases were reported, four additional cases of HCC with marked inflammatory cell infiltration have been documented as LEL-HCC in four articles [10-13], and the present case appears to be the tenth case reported in the English-language medical literature. The clinicopathological features of LEL-HCC have not been well documented, and even the criteria for diagnosing LEL-HCC are unclear. In this article, we used the term LEL-HCC for the present case and for the nine cases that were reported as LEL-HCC after Szekely initially proposed this disease entity. We also reviewed the clinicopathological features of these cases to further understand the disease.

In Table 1, we have summarized the clinicopathological features of the ten cases reported since the proposal by Emile et al. [9] and Szekely et al. [39]. There were eight males and two females, and the mean age was 55.0 years. Single tumors were reported in seven cases and multiple tumors in three cases. The mean diameter of the largest tumor was 30.9 mm. No metastases were found preoperatively in any of the cases except the present one, in which para-aortic lymph node metastasis was suspected prior to surgery. Only two patients had undergone chemoembolization before surgery. Six patients underwent liver transplantation, and four had surgical resection. Hepatitis B and C virus serology showed variable patterns, i.e., positive and negative in one case, negative and positive in three cases, and both positive in four cases. Seven patients had cirrhosis. Only one patient had serology positive for EBV. Infiltrating lymphocytes were T-cell dominant in nine cases [9,10,12,13], and the dominancy was not documented in one case [11]. T-cell markers investigated varied among the reports. In the five cases reported by Emile and associates, the mean CD3/CD20 ratio was 11/1 [9]. Si and colleagues investigated LCA, CD20, and CD3 in the infiltrating cells and described that there was a mixed lymphocytic population with an excess of T cells [10]. Nemolato and coworkers observed a predominance of CD8-positive lymphocytes in the tumor [12]. Park and associates reported that the infiltrating cells were predominantly CD4-positive lymphocytes [13]. While three patients died of HCC 39.5 months after surgery, the other seven were still alive after 55.3 months.

**Table 1:** Clinicopathological features of lymphoepithelioma-like hepatocellular carcinoma reported in the literature

**Case (ref.)**	**Age (year)s/sex**	**Tumor location**	**Number of tumors**	**Tumor size (mm)**	**Metastasis at surgery**	**Preoperative therapy**	**Surgery**	**Postoperative therapy**	**HBV**	**HCV**	**LC**	**EBV**	**Lymphocyte phenotype dominancy**	**Survival after surgery**
1 [9]	50/M	Not described	1	40	None	None	Liver transplantation	None	+	+	+	-	T cells	Alive without recurrence 10 years
2 [9]	54/M	Not described	2	20	None	Chemo-embolization	Liver transplantation	None	-	-	-	-		Dead of disease 7.7 years
3 [9]	59/M	Not described	4	50	None	None	Liver transplantation	Adjuvant chemotherapy	+	+	+	-		Alive without recurrence 8 years
4 [9]	45/M	Not described	1	20	None	None	Liver transplantation	Adjuvant chemotherapy	-	+	+	-		Alive without recurrence 4.7 years
5 [9]	64/M	Not described	2	40	None	Chemo-embolization	Liver transplantation	Adjuvant chemotherapy	-	-	+	-		Alive without recurrence 3 years
6 [10]	39/F	Not described	1	10	None	None	Liver transplantation	None	-	+	+	+	T cells	Dead of recurrence 5 months
7 [11]	56/M	Right lobe	1	30	None	None	Resection	Chemotherapy for recurrence	-	+	+	-	Not described	Dead of recurrence 21 months
8 [12]	47/F	Adjacent to gall bladder	1	22	None	None	Resection	Not described	-	-	-	-	T cells	Alive without recurrence 15 months
9 [13]	57/M	Hepatic dome	1	27	None	None	Resection	Not described	+	-	+	-	T cells	Alive without recurrence 50 months
10 (present case)	79/M	Lateral segment	1	50	Swollen para-aortic lymph node	None	Resection	Chemotherapy for recurrence	-	-	-	-	T cells	Alive with recurrence (para-aortic lymph nodes) 20 months

HBV: hepatitis B virus, HCV: hepatitis C virus, LC: liver cirrhosis, EBV: Epstein-Barr virus.

The prognosis of LEL-HCC is also an important consideration. Both Shirabe et al. and Wada et al. have suggested that HCC with lymphoid stroma has a favorable prognosis [14,15]. Our review showed that the prognoses varied among cases. Emile and associates stated that HCCs with lymphoid stroma had a better prognosis than those without, possibly due to an antitumor effect related to the lymphocytic infiltration [9]. Park and coworkers also reported a case of regressing HCC with massive lymphoid infiltration [13]. On the other hand, Si and associates described a patient with LEL-HCC who died 5 months after transplant surgery because of massive recurrence in the transplanted liver, suggesting that post-transplant immunosuppressive therapy played a role in the recurrence [10]. Chen and associates also reported a case of LEL-HCC in which the tumor was well encapsulated and did not have microvascular invasion, with a poor outcome [11]. Based on the data reported in the ten cases, we calculated the 1-year and 5-year survival rates as 90.0% and 77.1%, respectively. According to the Nationwide Follow-Up Survey of Primary Liver Cancer in Japan reported by the Liver Cancer Study Group of Japan, the 5-year survival rate of HCC patients who underwent resection was 54.2% [40]. Therefore, these findings suggest that the prognosis of LEL-HCC is more favorable than that of HCC. Further accumulation and analyses of cases are needed to determine the actual biological behavior of LEL-HCC.

We were also interested in determining whether a relationship exists between LEL-HCC and EBV. EBV is a herpes virus that infects the majority of the population before adolescence and persists for life in many individuals. The association of EBV with a variety of neoplasms, such as Burkitt’s lymphoma and nasopharyngeal carcinoma, has been well recognized. With regard to neoplasms in the hepatobiliary system, a case of LEL-CCC associated with EBV was first reported in 1996 by Hsu and colleagues [1]. Subsequently, it has been demonstrated that LEL-CCC cases with EBV positivity are relatively abundant [41]. On the other hand, only one case of EBV-positive LEL-HCC has been reported to date [10]. Although distribution of EBV-associated lymphoepithelioma-like carcinoma is restricted to foregut-derived organs, which include the liver, it is unlikely that an association exists between LEL-HCC and EBV with respect to tumorigenesis.

The biological implications of lymphoid infiltration in LEL-HCC are currently under debate, and the causes and consequences of this disease remain poorly understood. Immunohistochemical examinations in four of the five articles of LEL-HCC reported after 2000 revealed that the infiltrates were predominantly T-cells [9,10,12,13]. Cases reported before 2000 also described similar findings [14,15]. In our analysis, the inflammatory cells consisted of a mixture of both T-cells and B-cells in lymphoid stroma, but the infiltrates in the cancer nests were predominantly CD8- and TIA1-positive T-cells. Together, the findings in past reports and our observations suggest that there is a T-cell mediated cytotoxic response against some previous or concurrent events in LEL-HCC. One of the possibilities is that pretreatment by chemoembolization induces T-cell infiltration. However, there were only two patients with a history of pretreatment by chemoembolization, indicating that this hypothesis would not apply to all cases [9]. Hepatitis virus infection would affect the local inflammatory response, but it is difficult to make an association between hepatitis virus infection and lymphoid infiltration because this hepatitis viral infection is frequently seen in cases of HCC. It is also possible that the T-cell-dominant lymphoid infiltration represents a spontaneous immune response and a biological defense mechanism against cancer. This hypothesis supports the finding that LEL-HCC tends to have more favorable clinical outcomes as compared to HCC without lymphoid infiltration. Our patient was the second reported case in which there was neither a history of pretreatment by chemoembolization nor hepatitis virus infection. It is still not known how such a spontaneous immune response could occur in these two patients without any apparent trigger. The present case has unique characteristics, such as a past history of curative resection of extrahepatic bile ducts and pathological findings of atypical biliary epithelia suggestive of well-differentiated adenocarcinoma *in situ* in the resected specimen. The immunohistochemical examination (hepatocyte-positive, CK7- and CK19-negative HCC) suggested that the biliary carcinoma *in situ* and HCC develop from individual progenitor cells. It is very difficult to determine if an exposure to another coexistent cancer caused an oncological immune response in this case.

## Conclusion

In summary, since Szekely’s proposal in 2000, investigators have referred to HCC with heavy lymphoid infiltration as LEL-HCC in order to avoid confusion in the literature [39]. We comprehensively reviewed all nine cases that had been reported as LEL-HCC in the English-language literature together with the current case, and our results suggest that the prognosis of this disease entity is favorable and that it is not associated with EBV. It is likely that a T-cell mediated cytotoxic response is involved in the pathophysiology. It is possible that similar cases reported before 2000 also belong to this disease entity. Although definitive diagnostic criteria have not yet been established, it seems useful to refer to this disease entity as LEL-HCC. The small number of cases to date prevents a full description of this disease. Reporting additional cases and further investigations into biological characteristics of LEL-HCC are needed in order to generate appropriate diagnostic criteria and to clarify the significance and features of the associated lymphoid infiltration.

## Consent

Written informed consent was obtained from the patient for publication of the case report and accompanying images.

## Abbreviations

LELC: Lymphoepithelioma-like carcinoma; EBV: Epstein-Barr virus; LEL-CCC: Lymphoepithelioma-like cholangiocarcinoma; HCC: Hepatocellular carcinoma; LEL-HCC: Lymphoepithelioma-like hepatocellular carcinoma; CT: Computed tomography; MRI: Magnetic resonance imaging.

## Competing interests

The authors declare that they have no competing interest.

## Authors’ contributions

MS performed the operation as a chief surgeon and wrote the paper. YK participated in the writing of the paper. HT, YM, and MS analyzed the pathological specimens and wrote the pathological description. OI and SK supervised the writing of the paper especially in the Discussion section. AU and AT prepared the radiological images and wrote the radiological description. KM participated in the operation as an assistant. YA, HY, and MK prepared the data from the reviewed papers. MT participated in the operation as an assistant and supervised the writing of the paper. YK represents our surgical department and supervised the writing of the paper. All authors significantly contributed to this study and approved the final manuscript.
